# Expression of *tert* Prevents ALT in Zebrafish Brain Tumors

**DOI:** 10.3389/fcell.2020.00065

**Published:** 2020-02-11

**Authors:** Aurora Irene Idilli, Emilio Cusanelli, Francesca Pagani, Francesco Berardinelli, Manuel Bernabé, María Luisa Cayuela, Pietro Luigi Poliani, Maria Caterina Mione

**Affiliations:** ^1^Department of Cellular, Computational and Integrative Biology – CIBIO, University of Trento, Trento, Italy; ^2^Pathology, Department of Molecular and Translational Medicine, University of Brescia, Brescia, Italy; ^3^Department of Science, Roma Tre University, Rome, Italy; ^4^Telomerase, Cancer and Aging, Department of Surgery, Instituto Murciano de Investigación Biosanitaria-Arrixaca, Murcia, Spain

**Keywords:** telomeres, ALT, zebrafish, pediatric brain tumors, *tert*, TERRA, CO-FISH, C-circles

## Abstract

The activation of a telomere maintenance mechanism (TMM) is an essential step in cancer progression to escape replicative senescence and apoptosis. Alternative lengthening of telomeres (ALT) is found in a subset of malignant brain tumors with poor outcomes. Here, we describe a model of juvenile zebrafish brain tumor that progressively develops ALT. We discovered that reduced expression of *tert*, linked to a widespread hypomethylation of the *tert* promoter and increase in Terra expression precedes ALT development. Surprisingly, expression of *tert* during juvenile brain tumor development led to reduced proliferation of tumor cells and prolonged survival. Most importantly, expression of *tert* reverted all ALT features and normalizes TERRA expression, promoted heterochromatin formation at telomeres, and attenuated telomeric DNA damage. These data suggest that the activity of telomerase goes beyond telomere maintenance and has profound consequences on genome stability.

## Introduction

Telomeres are nucleoprotein structures assembled at the end of eukaryotic chromosomes protecting them from fusions, degradation, and erroneous recombination events. Critically short telomeres trigger a DNA damage response, ultimately leading to an irreversible cell cycle arrest, known as senescence (Harley et al., 1990). In order to attain unlimited proliferative capacity, cancer cells must adopt a telomere maintenance mechanism (TMM). Most cancers reactivate telomerase, which is usually expressed at very low levels in somatic cells (Kim et al., 1994). However, a minority of cancers use ALT to maintain telomere length, a mechanism based on homologous recombination (Dilley and Greenberg, 2015). Both the molecular details of ALT activation (Cesare and Reddel, 2010) and the specificity for certain tumors, either with mesenchymal (sarcoma) or neuroectodermal (glioblastoma) origin (Heaphy et al., 2011; Mangerel et al., 2014; Louis et al., 2016) remain to be defined. Notably, ALT activation can be inferred by a number of other features, including the presence of heterogeneous telomeres, with lengths ranging from very short to extremely long (Bryan et al., 1995; Cesare and Reddel, 2010), the presence of circular extrachromosomal telomeric repeats (ECTRs, containing partially single-stranded telomeric CCCTAA repeats, also known as C-Circles), increased telomeric recombination (detected by the presence of telomere sister chromatid exchange, T-SCE) and formation of complexes of promyelocytic leukemia nuclear bodies (known as ALT-associated promyelocytic leukemia (PML) bodies, APBs, Cesare and Griffith, 2004). APBs contain ALT-specific contents and proteins involved in DNA recombination/replication, and this content includes telomeric DNA, the telomere binding proteins TRF1 and TRF2, and factors implicated in DNA damage response and DNA double-strand break repair such as the Rad50/Mre11/NBS1 complex, Rad51/Rad52 and the replication factors (RPA) (Henson et al., 2002). Despite their heterochromatic nature, telomeres produce TERRA, a lncRNA playing an important role in telomere stability (Azzalin et al., 2007; Schoeftner and Blasco, 2008). TERRA transcripts are required for proper telomeric DNA replication in telomerase-positive human cancer cells (Beishline et al., 2017) and in ALT cancer cells, TERRA can promote homologous recombination between chromosome ends (Arora et al., 2014). Despite the established impact of telomere biology in cancer and the recent advancements on the role of chromatin structure and TERRA in TMMs in cancer, the molecular events that trigger ALT in a developing tumor remain unclear. The study of ALT in cancer has mostly relied on ALT sarcoma cell lines (Heaphy et al., 2011) and their telomerase positive counterparts, and only recently TMMs started to be evaluated in primary cancers (Pompili et al., 2017). Mouse models have been used extensively to study telomere biology (Goytisolo and Blasco, 2002) and have enabled essential progresses in the field. However, mouse telomeres are considerably longer than human telomeres (50 kb vs. 15 kb, Calado and Dumitriu, 2013) and telomerase continues to be expressed in most adult mouse tissues and organs, with the result that telomere shortening is not a problem for cellular lifespan in mouse. By contrast, zebrafish telomeres (15–20 kb) are relatively similar to human telomeres (10–15 kb) and, although telomerase is constitutively active in some organs, the expression of *tert* mRNA, the catalytic subunit of telomerase, telomerase activity and telomere length decrease drastically with age, similarly to human tissue; in addition, *tert* levels in the zebrafish brain are extremely low (Xie et al., 2008; Anchelin et al., 2011, 2013).

Recently, we generated a zebrafish model of brain tumor based on somatic expression of human oncogenes. In this model, brain tumors resemble the molecular mesenchymal subtype of glioblastoma (Mayrhofer et al., 2017). Here, we investigated the TMM adopted by juvenile zebrafish brain tumors and found that they progressively develop ALT. The activation of ALT is preceded by a reduction of telomerase expression and a significant increase of TERRA levels. Surprisingly, *tert* re-expression prevents the development of ALT and promoted heterochromatin formation, partially reducing oncogene-induced DNA damage at telomeres. Most importantly, tert re-expression, reduces proliferation and malignity of brain tumors and extended survival of fish with juvenile brain cancer, suggesting that *tert* controls telomere stability in these tumors.

## Materials and Methods

### Maintenance of Zebrafish and Line Generation

Adult zebrafish (*Danio rerio*) were housed in the Model Organism Facility – Center for Integrative Biology (CIBIO) University of Trento and maintained under standard conditions (Westerfield et al., 2009). All zebrafish studies were performed according to European and Italian law, D.Lgs. 26/2014, authorization 148/2018-PR to MM. Fishes with somatic and germline plasmid expression were generated as describe (Santoriello et al., 2010; Mayrhofer et al., 2017).

The following zebrafish transgenic lines were used or generated in the course of this study:

*Et(zic4:Gal4TA4, UAS:mCherry)_*hzm*__5_* called zic:Gal4 (Mayrhofer et al., 2017).
*Et(kita:Gal4TA4, UAS:mCherry)_*hzm*__1_* called Kita:Gal4 (Santoriello et al., 2010).
*Tg(UAS:eGFP-HRAS_G12V)_*io*__006_* called UAS:RAS (Santoriello et al., 2010).
*hu3430 (Tert-/-)* (Anchelin et al., 2011).
*Tg(10xUAS:tert)* this study.
*Tg(10xUAS:terc)* this study.

### Cell Culture and Cell Lines

The U2OS, HeLa, L5178Y-S, and L5178Y-R lymphocyte cell lines were cultured in Dulbecco’s modified Eagle’s medium (DMEM) supplemented with 10% (v/v) fetal bovine serum (FBS) in a humidified incubator at 37°C with 5% CO_2_. Cell lines were tested regularly for mycoplasma contamination by Celltech CIBIO facility.

### DNA Constructs and Transgenic Line Generation

The genes encoding zebrafish terc (ENSDARG00000042637.10) and tert (ENSDARG00000042637.10) were synthesized and cloned in pBluescript II KS+ and subcloned in a pEntry vector (pME-MCS Tol2Kit)^1^. The UAS:terc; cmlc2:eGFP and UAS:tert; cry:eGFP constructs were generated by MultiSite Gateway assemblies using LR Clonase II Plus (Invitrogen) according to standard protocols, and Tol2kit vectors described previously (Kwan et al., 2007). 25 pg of the final construct and 25 pg of tol2 transposase mRNA were injected into 1-cell stage embryos and founder fish (F0) for terc or tert were identified based on green fluorescent heart or fluorescent eyes. Embryonic brain expression was obtained by outcrossing them with the zic:Gal4 line.

### Terminal Restriction-Fragment

Telomere length assay was performed as described in Kimura et al. (2010). Briefly, genomic DNA was extracted from freshly isolated zebrafish brains following the instruction of ReliaPrep^TM^ gDNA Tissue Miniprep System (Promega Corporation), and then 3 μg of genomic DNA were digested with *Rsa*I and *Hin*fI enzymes (New England Biolabs) for 12 h at 37°C. After electrophoresis, the gel was blotted and probed with antisense telomere probe (CCCTAA)8 labeled with DIG Oligonucleotide 3′-End labeling Kit (Roche) or with a 1.6 kb fragment containing the sequence (TTAGGG)n (Hanish et al., 1994; Kimura et al., 2010) labeled with Nick Translation Kit (Roche) following instructions manual. Image Lab^TM^ Software (Bio-Rad) was used to analyse telomere length from TRF analysis, and data were plotted using GraphPad Prism.

### Quantitative Fluorescence *in situ* Hybridization (Q-FISH) on Interphase Nuclei

Quantitative fluorescence *in situ* hybridization (Q-FISH) on interphase nuclei was performed as described in Canela et al. (2007). Briefly, cell suspensions were obtained by pipetting up and down zebrafish brains in 500 μL of ice-cold 0.9 × PBS with 10% FBS and subsequently filtered through a 100 and 40 μm mesh. Cell suspensions were then centrifuged at 250 × *g* for 8 min at 4°C. The supernatant was decanted, and cells were incubated for 25 min at 28.5°C in a hypotonic solution (1.1% sodium citrate). Cells were recovered after centrifugation and fixed in ice-cold Carnoy’s methanol: glacial acetic acid fixative (3:1, v:v). Cells were then dropped onto wet superfrost microscope slides and allowed to dry overnight. After rehydration in PBS 1X, slides were fixed in 4% paraformaldehyde (PFA, Invitrogen) for 2 min and washed three times for 5 min in PBS 1×. Slides were treated 10 min at 37°C with 1 mg/ml HCl-pepsin (pH 2, Sigma). After a step of PFA fixation and PBS 1× washing, slides were dehydrated in ethanol series and air-dried. Slides were then hybridized 3 min at 80°C and 2 h at RT in wet chamber with 20 μl per slide on coverslip of 0.3 μg/ml PNA probe TelC-Cy3 (Panagene) in hybridization buffer (70% formamide, 0.25% blocking reagent, 10 mM Tris (pH 7.2), 1× Buffer MgCl_2_); Slides were then washed two times for 15 min in 70% formamide, 10 mM Tris (pH 7.2), 0.1% BSA and three times in TBS 1×. Slides were dehydrated with ethanol series, air dried, and mounted with glycerol antifade solution [90% glycerol, 5 mg/mL n-propyl gallate, 0.1M Tris (pH 9.0) containing 0.2 μg/ml of 4’-6-diamidino-2-phenylindole (DAPI)]. Z stack images were acquired with an inverted Leica DMi8 fluorescent microscope equipped with a monochromatic Andor Zyla 4.2 Megapixel sCMOS camera, using an HC PL Apo CS2 63×/1.4 oil immersion objective. Z-stack images were processed to remove background using Fiji/ImageJ and then telomere fluorescence signals were quantified using the TFL-TELO program (from Peter Lansdorp, Vancouver, BC, Canada). Data were plotted using GraphPad Prism.

### Telomerase Activity Assay

Real-time quantitative TRAP (Q-TRAP) assay was performed as described in Anchelin et al. (2011). Protein extracts were obtained adding 200 μL of 1× CHAPS to dissociated control brains (Ctrl and tert-/-) and brain tumors (RAS) and incubate on ice for 30 min. The hu3430 tert-/- mutant strain was used here. After sample lysate centrifugation (16,000 × *g* for 20 min at 4°C), total protein concentration was measured using a BCA protein assay kit (Pierce) according to the manufacturer’s protocols. 1 μg of protein extract was used to perform Q-TRAP. A master mix was prepared with 1× SYBR Green Master Mix (Resnova – PCR Biosystem), 100 ng TS primer per sample (5′-AATCCGTCGAGCAGAGTT-3′), 100 ng ACX primer per sample (5′-GCGCGGCTTACCCTTACCCTTACCCTAACC-3′), 1 mM EGTA and RNase/DNase-free water to a final volume of 25 μL. 2 μL of sample were added to 23 μL master mix in a 96-well PCR plate and incubate 30 min at 30°C in the dark for extension of telomerase substrate. Real-time PCR was performed with a CFX96 Real-Time PCR Detection System (Bio-Rad) machine using the standard protocol: 95°C for 10 min; 40 cycles at 95°C for 15 s and at 60°C for 60 s. In all cases, each PCR was performed with triplicate samples and repeated with at least two independent samples. As a negative control, the 1 μg of protein assayed of each sample extract was incubated with 1 μg of RNAse A (QIAGEN) at 37°C for 20 min. A 1:10 dilution series of telomerase-positive sample (HeLa) was used for making the standard curve. After PCR, real-time data were collected and converted into relative telomerase activity (RTA) units based on the following formula: RTA of sample = Delta10^(Ct sample–Yint)^/slope. Q-PCR analysis was performed with Microsoft Excel and GraphPad Prism.

### Analysis of Gene Expression

Total RNA was extracted from larval heads and brains/tumors with TRIzol reagent (Invitrogen). Total RNA was cleaned up using RNeasy Mini Kit (Qiagen) following the manufacturer’s instructions and treated twice with DNase I (1 unit/μg RNA, Qiagen). The RNA concentration was quantified using nanodrop2000 (Thermo Fisher Scientific) and VILO superscript KIT (Thermo Fisher Scientific) was used for First-strand cDNA synthesis according to the manufacturer’s protocol. qRT-PCR analysis was performed using qPCR BIO Sygreen Mix (Resnova – PCR Biosystem) using a standard amplification protocol. The primers used for zebrafish tert were: forward 5′-CGGTATGACGGCCTATCACT-3′ and reverse 5′-TAAACGGCCTCCACAGAGTT-3′; for 3′ UTR zebrafish tert were: forward 5′-AACACTTGATGGTGACTGT-3′ and reverse 5′-GACTTCTGCATCGATCTGTGAT-3′; for zebrafish rps11: forward: 5′-ACAGAAATGCCCCTTCACTG-3′ and reverse: 5′-GCCTCTTCTCAAAACGGTTG-3′; for human 36B4: forward 5′-CAGCAAGTGGGAAGGTGTAATCC-3′ and reverse: 5′-CCCATTCTATCATCAACGGGTACAA-3′. To determine terc and TERRA levels, total RNA (1 μg) was treated an additional time with TURBO^TM^ DNase (2 unit/μg RNA, Ambion) and then reverse transcribed with gene-specific primers (zebrafish terc: 5′-TGCAGGATCAGTGTTTGAGG-3′; rps11: 5′ -GCCTCTTCTCAAAACGGTTG-3′; Telo RT 5′-CCCTAACCCTAACCCTAACCCTAACCC TAA-3′, human 36B4 RT 5′-CCCATTCTATCATCAACGGGTACAA-3′) using Superscript III (Thermo Fisher Scientific) at 55°C for 1 h, followed by RNase H treatment. qRT-PCR analysis was performed using qPCR BIO Sygreen MIx (Resnova – PCR Biosystem) with 500 nM specific primers (zebrafish terc forward: 5′-GGTCTCACAGGTTTGGCTGT3′, reverse 5′-TGCAGGATCAGTGTTTGAGG-3′); (zebrafish TERRA forward: 5′-CGG TTT GTT TGG GTT TGG GTT TGG GTT TGG GTT TGG GGT-3′, reverse 5′-GGC TTG CCT TAC CCT TAC CCT TAC CCT TAC CCT TAC CCT-3′). rps11 and 36B4 specific primers were used as zebrafish and human reference. The amplification program was as follows: 95°C for 10 min followed by 36 cycles at 95, 58, and 72°C each for 10 s. Real-time PCR was performed with a CFX96 Real-Time PCR Detection System (Bio-Rad) machine. Q-PCR analysis was performed with Microsoft Excel and Graphpad Prism. In all cases, each PCR was performed with triplicate samples and repeated with at least two independent samples. Reactions without reverse transcriptase were performed as controls for TERRA quantification.

### Zebrafish 5mC *tert* Promoter ChIP Protocol

Larval heads and brains/tumors samples were incubated for 8 min at room temperature in 500 μl 1% formaldehyde in PBS+ protease inhibitors. Then 50 μl of Glycine 1.25M were added to stop cross-linking reaction, samples were briefly vortexed and incubated for 5 min at room temperature on a wheel. Upon centrifugation, pellets were washed in 1.2 ml ice-cold PBS, resuspended in 500 μl of PBS and homogenized using a homogenizer. After homogenization, samples were centrifuged 5000 rpm for 10 min at 4°C and pellets resuspended in 600 μl of Lysis buffer (50 mM Tris pH 8; 10 mM EDTA pH 8; 1% SDS) + protease inhibitors and incubated 10 min in ice. Samples were vortexed for 30 s then centrifuged 1000 rpm for 1 min at 4°C. The supernatant was collected into a new tube while the pellet was resuspended in 300 μl of Lysis buffer + protease inhibitors. After vortex and spin, as before, the supernatant was collected and added to the previous 600 μl of sample making a 900 μl lysate for each sample. The lysate was then divided into three tubes, and sonication performed using the Bioruptor instrument (DIAGENODE) with the following setting: 4 x cycles 20 s ON and 60 s OFF at 4°C. After sonication, 8 μl of lysate are run on agarose gel to verify chromatin shearing. 300–600 nt long DNA fragments are expected. Sonicated samples were centrifuged 5000 rpm for 5 min at 4°C. The supernatant was collected in a new tube. At least 100 μg of proteins were used for subsequent IP. One-tenth of the amount of proteins used for IP is collected for the INPUT and frozen at −80°C. 3.6 ml of dilution buffer (20 mM Tris pH 8; 150 mM NaCl; 2 mM EDTA pH 8; 1% Triton) were added to each sample to be immunoprecipitated. A pre-clearing step was performed by adding 40 μl of protein G Dynabeads for 2 h at 4°C on a wheel. Upon Dynabeads removal each sample was then divided into two new tubes, in which 2 μg of either anti-5mC (Abcam) or IgG (Abcam) were added. Antibody incubation was performed overnight at 4°C on a wheel. The following morning, 20 μl of pre-equilibrated protein G-Dynabeads were added to each IP and samples were incubated at 4°C for 2 h on a wheel. For each sample, Dynabeads-chromatin complexes were recovered using magnetic rack and washed three times with 1 ml wash buffer and one time with the final wash buffer. Each wash was performed for 5 min at room temperature. Then 450 μl of elution buffer (1% SDS; 0.2M NaCl; 0.1M NaHCO_3_) + 18 μl of NaCl 5M solution + 5 μl of RNAse A solution (10 mg/ml solution) were added to each sample. Samples were vortexed vigorously and incubated at 37°C for 1 h. Then 250 μg of proteinase K was added to each sample and incubated at 65°C overnight. The following morning DNA was extracted using phenol/chloroform protocol, precipitated with Ethanol 100%-Sodium-Acetate solution in the presence of 20 μg glycogen, washed in Ethanol 70%, air-dried and resuspended in 30 μl (IP) water. Every step was performed also on the INPUT.

### C-Circles Assay

C-Circles assay was performed as described (Henson et al., 2017). Briefly 30 ng of genomic DNA was combined with 0.2 mg/ml BSA, 0.1% (v/v) Tween20, 1 mM each dATP, dTTP, dGTP, 4 μM dithiothreitol (DTT), 1×Φ29 DNA polymerase buffer, 7.5 U Φ29 (New England Biolabs). Rolling circle amplification (RCA) reaction was performed by incubation at 30°C for 8 h, plus 20 min at 65°C. Reactions without the addition of Φ29 polymerase were included as a control (“–Φ29”). For dot blot detections, the CCA products (plus 40 μl 2× SSC) were dot-blotted onto 2× SSC-soaked positive nylon membrane, thanks to a 96-well Bio-Dot Microfiltration Apparatus (Bio-Rad). The membrane was UV-crosslinked for 3 min/each side and hybridized with probe (CCCTAA)8 labeled with DIG Oligonucleotide 3′-End labeling Kit (Sigma-Aldrich) and developed as described (Henson et al., 2017). Image Lab^TM^ Software (Bio-Rad) was used to analyse dot intensity. The result of the C-Circle assay dot blot was evaluated according to Henson et al. (2017). Q-PCR detection was performed as described (Lau et al., 2013). Briefly, CCA products were diluted four times in water and used as templates in a qPCR using telomF (300 nM) 5′-GGTTTTTGAGGGTGAGGGTGAGGGTGAGGGTGAGGGT-3′ and telomR (400 nM) 5′-TCCCGACTATCCCTATCCCTAT CCCTATCCCTATCCCTA-3′ primers. qPCRs using rps11 primers (150 nM) and 36B4 primers (Forward 300 nM and Reverse 500 nM) were performed for single copy gene (SCG) normalization in zebrafish and human samples, respectively. All qPCRs were done in triplicates. Each telomere Ct was normalized with the SCG Ct (normTEL). The telomere content (TC) of a sample was the normTEL value in the –Φ29 reactions. The CC abundance of a sample was calculated as (normTEL in + Φ29) – (normTEL in –Φ29). ALT activity was considered significant if at least twice than the levels without Φ29 polymerase.

### TERRA Dot-Blot

Before blotting, 500 ng of total RNA (in 1 mM EDTA, 50% formamide – Volume 100 μl) were denatured in a thermocycler at 65°C for 10 min and then on ice. As control for DNA contamination, we treated 500 ng of total RNA from each sample with RNAse A (0.2 mg/ml RNAse A, Sigma-Aldrich, for 30 min at 37°C) prior denaturation. Denaturated RNA was dot-blotted onto 2× SSC-soaked positive nylon membrane and then UV crosslinker for 3 min/each side. Hybridization was performed at 50°C O/N with the probe 1.6 kb fragment containing the sequence (TTAGGG)n (Hanish et al., 1994) labeled with Nick Translation Kit (Sigma-Aldrich) and developed as described above according to Kimura et al. (2010). Image Lab^TM^ Software (Bio-Rad) was used to analyse dot intensity; quantitative analysis of dot blot intensity was performed after background subtraction and on control normalization. Data were plotted GraphPad Prism. A digoxigenin-labeled actin mRNA sense probe, obtained from *in vitro* transcription, was used as loading control.

### TERRA RNA-FISH

Cell-derived from zebrafish brains and tumor brains were seeded on poly-D Lysine (1 μg/mL) (Sigma-Aldrich) slides 1 h before starting the experiment. TERRA RNA–FISH assay was performed as described (Arnoult et al., 2012). Z-stack images were acquired with an inverted Leica DMi8 fluorescent microscope equipped with a monochromatic Andor Zyla 4.2 Megapixel sCMOS camera, using an HC PL Apo CS2 63×/1.4 oil immersion objective. Z-stack images were processed for removing background using Fiji/ImageJ and fluorescence signals were quantified using the TFL-TELO program (from Peter Lansdorp, Vancouver, BC, Canada). Data were plotted using GraphPad Prism.

### Two-Color Chromosome Orientation FISH (CO-FISH) in Metaphase Spreads

Metaphase spreads were obtained from larval heads and brains/tumors from adult individuals. For larvae: 30 hpf embryos, previously dechorionated, were incubated with BrdU 10 mM//BrdC 4 mM (Alfa Aesar^TM^), 1% DMSO for 6 h at 28°C and then with 1 μg/μl Nocodazole (Sigma-Aldrich) for an additional 6 h before preparing cell suspensions. For adults, 5 μl of BrdU/C and Nocodazole (at the concentration reported above) were retro-orbital injected (Pugach et al., 2009) with 12 h interval; fishes were processed 24 h after the first injection. Cell suspensions were obtained as previously described. Cells were then incubated for 25 min at 28.5°C in a hypotonic solution (2.5 g/L KCl, in 10 mM HEPES, pH 7.4) and fixed in ice-cold Carnoy’s methanol: glacial acetic acid fixative (3:1, v:v) per 2 h. After a wash in Carnoy’s methanol, cells were dropped onto superfrost microscope slides (pre-cleaned and wet) and allowed to dry overnight at room temperature in the dark. Degradation of newly synthesized strand and 2-Color FISH was performed as described (Arnoult et al., 2008). Metaphase spread chromosomes were counterstained with DAPI. Z-stack images images were acquired with an inverted Nikon Ti2 fluorescent microscope equipped with a monochromatic Andor Zyla PLUS 4.2 Megapixel sCMOS camera, using a Plan Apochromatic 100×/1.45 oil immersion objective. Images were processed for background subtraction using Fiji/ImageJ.

### Immunostaining on Paraffin-Embedded Sections

Briefly, 2-μm-thick paraffin sections were deparaffinized and rehydrated. Endogenous peroxidase activity was blocked with 0.3% H_2_O_2_ in methanol for 20 min. Antigen retrieval (when necessary) was performed in either 1.0 mM EDTA buffer (pH 8.0) or 1 mM Citrate buffer (pH 6.0). Sections were then washed in TBS (pH 7.4) and incubated primary antibodies diluted in TBS 1% BSA at 4°C overnight. The antibody used and their dilutions were as follows: PCNA 1:200 (Santa Cruz Biotechnology, cat. No. SC-25280); GFAP 1:1000 (Dako, cat. No. 20334). For immunofluorescence, secondary antibodies conjugated with FITC and/or Texas Red was used for 2 h at room temperature, and nuclei were counterstained with DAPI. Images were acquired using an inverted Nikon Ti2 fluorescent microscope equipped with a monochromatic Andor Zyla PLUS 4.2 Megapixel sCMOS camera. Images were processed for background subtraction using Fiji/ImageJ.

### Immunofluorescence Combined With Q-FISH

Cell suspensions derived from zebrafish brain tumors were seeded on Poly-lysine (1 μg/mL) (Sigma-Aldrich) slides. Briefly, after 1 wash in TBS 1× per 5 min, slides were fixed in 2% paraformaldehyde containing 2% sucrose for 10 min at RT and then washed twice in TBS, followed by permeabilization with 0.5% Triton for 15 min. After three washes in TBS, slides were incubated 1 h at RT in blocking buffer (5% NGS, 0.1% Triton for H3K9me3 or 0.5% BSA, 0.2% Gelatin cold water fish skin in 1× PBS for γH2AX) and then overnight at 4°C with primary antibody with the following dilutions: γH2AX 1:300 (Millipore, cat. No05-636), H3K9me3 1:500 (Abcam, cat no. ab8898). After three washes in blocking buffer, slides were incubated with secondary antibody (goat-anti-mouse 488 or Goat-anti-rabbit 488 – Thermo Fisher Scientific) 1:500 for 2 h at RT. After incubation, slides were washed three times in 1× TBS (5 min each) and one time in Q-FISH washing buffer (0.1%BSA, 70% formamide, 10 mM Tris pH 7.2). Then FISH was performed with PNA TelC-Cy3 probe (PANAGENE) as described above. Nuclei were counterstained with DAPI. Z-stacks Images were captured at 100× magnification (Plan Apochromatic 100×/1.45 oil immersion objective) using an inverted Nikon Ti2 fluorescent microscope equipped with a monochromatic Andor Zyla PLUS 4.2 Megapixel sCMOS camera. Images were processed for background subtraction using Fiji/ImageJ. Colocalization analysis was performed with DiAna^2^ (ImageJ) 65, calculating co-localization between objects in 3D, after 3D spot segmentation.

### Statistics

All the graph and the statistical analysis (Mann–Whitney – non-parametric test, no Gaussian distribution, two-tailed, interval of confidence: 95%) were generated and calculated using GraphPad Prism software version 5.0. For all experiments a minimum of three fish or groups per genotype was used, unless differently specified. Details regarding number of samples used and statistical analysis are provided in the figure legends.

## Results

### Telomeres in Zebrafish Brain Tumors Are Heterogeneous in Length

**FIGURE 1:**
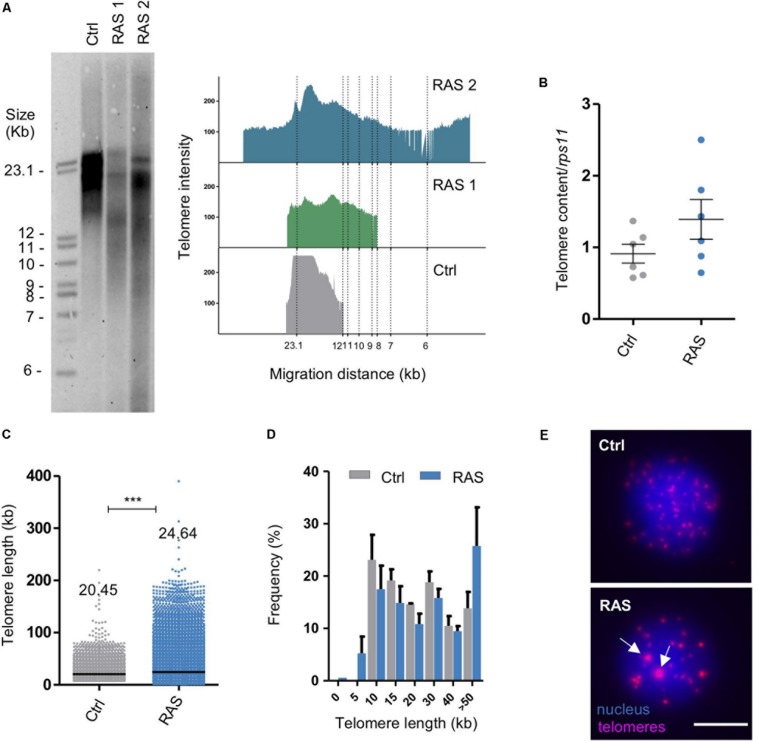
Zebrafish brain tumors have heterogeneous telomeres. **(A)** Telomere length analysis via TRF in one control and two RAS tumors. The panel on the right shows TFR analysis obtained by graphing intensity of the signal versus DNA migration. **(B)** Relative telomere content determined by telomere qPCR and normalized to the signal of a single copy gene (*rps11*) in controls (Ctrl, *n* = 7 brains) and RAS tumors (RAS, *n* = 7 brains). Bars represent mean ± SEM. **(C)** Quantification of relative telomere length measured by Q-FISH and given a kb value based on the fluorescent intensity of L5178Y-S and L5178Y-R lymphocyte cells with known telomere lengths of 10.2 and 79.7 kb, respectively. See also Supplementary Figure 1B. Number of telomeres examined: Ctrl *n* = 3027, RAS = 9738. Data from three independent experiments were combined. Median values are reported on the graph. ***p < 0.0001. Scatterplot bars represent median. **(D)** Distribution of telomere length evaluated by Q-FISH in Control (gray *n* = 2) and RAS tumors (light blue *n* = 3). The very long telomeres (>30 kb) could represent telomeric clusters, an ALT feature. **(E)** Representative fluorescent microscope images of Q-FISH analysis of control and RAS nuclei with ultrabright foci (white arrows). Scale bar: 5 μm. TRF: telomere restriction fragment; Ctrl: control; RAS: brain tumors. See also Supplementary Figure 1.

In order to investigate telomere length in zebrafish brain tumors we used 1–2 month old zebrafish with RAS-induced brain tumors (Mayrhofer et al., 2017), here called RAS, and performed terminal restriction fragment (TRF) analysis followed by Southern blot. Telencephalic RAS brain tumors were compared with similar age telencephalic tissue from control (wild type) fish. RAS brain tumors showed highly heterogeneous telomere lengths as observed by the smeared bands detected on the TRF blot (Figure 1A). The percentage of RAS-induced brain tumors with lenght heterogeneity is 66.7% (observed in 6/9 TRF). In these samples, telomeres ranged from less than 6 kb to more than 23 kb in size, whereas control brains showed a more compact band around 20 kb (Figure 1A and Supplementary Figure 1A). Notably, telomere lengths of RAS brain tumors varied between samples and highly resemble the variations in the length of telomeres detected in U2OS cells (Supplementary Figure 1A), which are known ALT positive human cancer cells.

In order to confirm these results, we performed telomere qPCR analyses using genomic DNA extracted from RAS tumor samples or control brains. These experiments revealed an increase of telomere content in the RAS samples (Figure 1B), which is consistent with the results obtained from the TRF experiments. We then sought to evaluate differences in telomere length distribution at single-cell resolution, by performing Q-FISH experiments using fluorescently labeled telomere specific probe. In these analyses, we included as calibrators of single-telomere fluorescence intensity the L5178Y-S and L5178Y-R lymphocyte cell lines, which have telomere lengths of approximately 10 and 79 kb, respectively (Canela et al., 2007; Supplementary Figures 1B,C). Q-FISH experiments revealed a median telomeric length of 20.45 kb for control brains and 24.64 kb for tumor samples (Figure 1C). Interestingly, single-telomere fluorescence intensity distribution revealed the presence of very short telomeres (less than 5 kb) and very long telomeres or clusters (more than 50 kb) only/preferentially in tumor cells as compared to control cells (Figure 1D). The presence of clustered telomeres was confirmed by the occurrence of ultra-bright telomeric foci (Figure 1E, lower panel white arrows).

Overall, these results indicate that brain tumor cells have highly heterogeneous telomeres as observed by TRF, telomere qPCR and Q-FISH analyses. Thus brain tumor cells may have activated a TMM, which contrasts the progressive telomere shortening of control brain cells (Anchelin et al., 2011).

### Telomerase Is Not Involved in TMM in Zebrafish Brain Tumors

**FIGURE 2:**
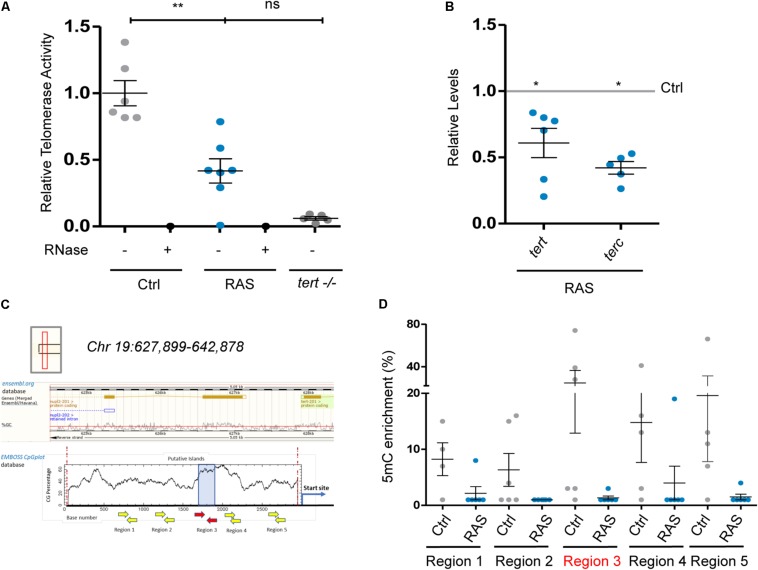
Telomerase is not involved in telomere maintenance in zebrafish brain tumors. **(A)** Relative telomerase activity measured by Q-TRAP in control, RAS and tert-/- brains, using 1 μg of protein extracts. RNase treatment (+) was used as a negative control to confirm the specificity of the assay, *n* = 6; **p = 0.005. **(B)** Expression of zebrafish *tert* and *terc* mRNA in brain tumors measured by RT-qPCR. Values were normalized to *rps11* mRNA levels and are relative to *tert* and *terc* expression in control brains (gray line set at 1.0) *n* = 6; *p < 0.05. **(C)** Schematic representation of the genomic region harboring putative CpG island on the tert promoter, according to Ensemble (upper panel) and EMBOSS CpG plot (lower panel) databases. Position of primers used in ChIP experiments is shown as arrows. Red arrows show primers amplifying a putative CpG island. **(D)** qPCR analysis of DNA CpG Methylation (5-methylcytosine) status of the tert promoter in control and brain tumors of 2 month old fishes. Different regions of the promoter were analyzed, red arrows indicate the position and primers for a putative CpG island. Values were normalized first to *rps11* and then to 5mC enrichment, with IgG enrichment set at 1.0. Bars in a, b, e represent mean ± SEM. *n* = 4–6. Ctrl: control brain, RAS: brain tumor.

Telomerase activity is tightly regulated during development and is re-activated in many tumors, where it is a critical determinant of cancer progression. To understand if the telomere length heterogeneity detected in zebrafish brain tumors is dependent on telomerase activity, we performed a quantitative telomeric repeat amplification protocol (Q-TRAP) to test the activity of the telomerase holoenzyme. By this analysis, we found a 2.5-fold reduction in telomerase activity in brain tumors (Figure 2A) compared to control brains of individuals of the same age. We next quantified the expression of the two components of the telomerase holoenzyme, the catalytic subunit, *tert*, and the template RNA, *terc*, through RT-qPCR (Figure 2B). *Tert* and *terc* transcript levels were significantly reduced in tumors (*tert* was reduced 1.7-fold; *terc* was reduced 1.9-fold) compared to control brains. To gain insight into the possible mechanism of *tert* downregulation, we analyzed the DNA methylation status of the zebrafish tert promoter in several brain tumors by performing chromatin immunoprecipitation (ChIP) of 5-methylcytosine (5mc) enriched DNA sequences. Using EMBOSS CpGplot^3^, we identified five CG-rich regions in the *tert* promoter (GRCz11, chr: 19: 627,899–642,878)^4^, one of which is predicted to harbor a CpG island in position –1849 from the start of the gene coding sequence (Figure 2C, red arrows). We examined the methylation status of these five *tert* promoter regions in both control and brain tumors. We found that a general pattern of hypomethylation of the *tert* promoter was present in tumor samples as compared to control samples (Figure 2D). Each of the six tumors analyzed showed the same hypomethylated promoter status. The association between promoter hypomethylation and low expression has also been reported for human *TERT* in brain cancer (Deeg et al., 2017). This relation suggests that promoter hypomethylation is a general feature of negative regulation of *tert* expression, perhaps due to the location of the gene in a subtelomeric region (CHR5:1253147-1295069, GRCh38/hg38), both in human and in zebrafish (Figure 2C, inset). In summary, these results indicate that expression of *tert* is reduced in brain tumors, and *tert* reduction associates with a hypomethylation status of the *tert* promoter and correlates with a significant decrease in the activity of telomerase. Overall, these findings suggest that telomeres in zebrafish brain tumors are not maintained by telomerase.

### Zebrafish Brain Tumors Have ALT Features

**FIGURE 3:**
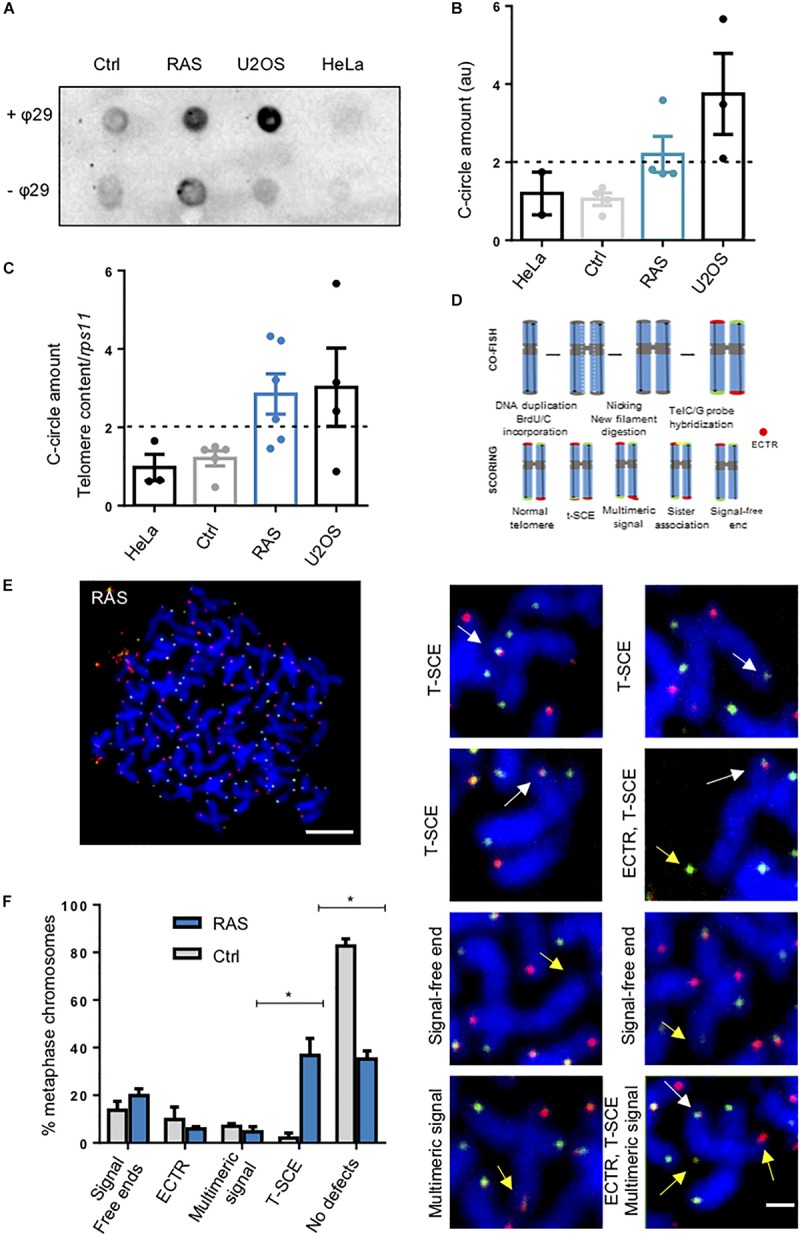
Zebrafish brain tumors are ALT. **(A)** Representative C-Circle assay by dot blot in one control and one brain tumor compared with telomerase positive HeLa cells and ALT U2OS cells. Reactions without phy29 polymerase (–θ29) were included as a control. **(B)** Quantitation and analysis of 4 C-Circle assay dot blot. Determination of ALT amount was calculated after subtracting global background and specific –θ29 signal. a.u.: arbitrary unit. **(C)** C-Circle assay quantified by telomere qPCR. Data are represented as amount of C-circles, normalized to telomere content (TC) and single copy gene (*rps11*). HeLa and U2OS were added as a reference. Ctrl *n* = 5; RAS *n* = 7. The dashed line indicates the level above which ALT activity is considered significant. Whiskers box plots represent median: min to max values. **(D)** Schematic drawing to describe the procedure for 2-color CO-FISH and the interpretation of telomere status based on the signals. **(E)** Two-color CO-FISH of a representative metaphase nucleus derived from a RAS brain tumor cell (scale bar 5 μm). The right panels **(D)** show details of telomeres with T-SCE (white arrows), signal free ends, multimeric signal and/or ECTR (yellow arrows) (scale bar 1 μm). **(F)** Quantitation of telomeric defects revealed in RAS brain (*n* = 88 chromosomes) compared with Control brain (*n* = 56 chromosomes). T-SCE: Telomere Sister Chromatid Exchange; ECTR: Extra-Chromosomal Telomeric Repeat; CO-FISH: Chromosome orientation FISH. Multiple t-test, Holm Sidak methods, *p < 0.05. Bars represent mean ± SEM. See also Supplementary Figure 2.

Next, we investigated whether ALT markers can be detected in tumors samples. We employed the C-Circle assay (CCA) to investigate the presence of C-Circles in zebrafish control and tumor samples, and, as additional controls, in two human cell lines, U2OS, and ALT cancer cell line, and HeLa, a telomerase-positive cell line (Henson et al., 2009, 2017). In these analyses, CCA products were detected by telomeric dot blot (Figure 3A) and telomeric qPCR (Figure 3B). Both methods showed that most of the tumors analyzed (10/13) were positive for C-Circles, with some variations in the levels of CCA products. Confirming the reliability of our approach, the amount of C-Circles detected in zebrafish tumor samples was similar to the amount of C-Circles detected in U2OS cells, while no positive signal was detected in HeLa or control brain samples.

CO-FISH experiments (Figure 3C) revealed the occurrence of T-SCEs in RAS brain cells (Figures 3D,D’, white arrows). Moreover, the same analyses revealed the presence of telomeric defects, such as multimeric signals, signal-free ends and ECTRs (Figures 3D,D’ and Supplementary Figure 2, yellow arrows), on mitotic chromosomes of cells derived from zebrafish brain tumors (see also Figure 3E).

**FIGURE 4:**
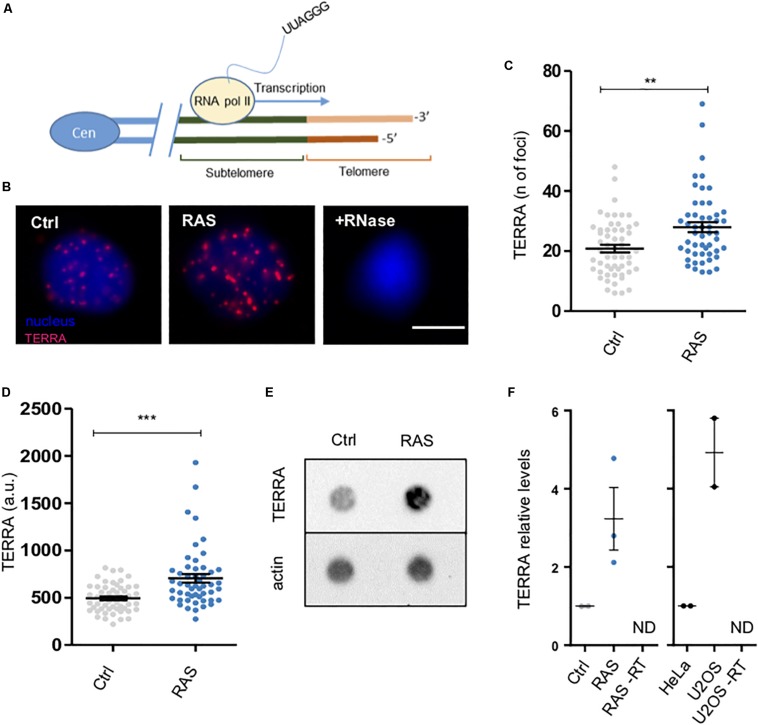
Zebrafish brain tumors exhibit elevated TERRA expression. **(A)** Schematic drawing describing the generation of TERRA from subtelomeric regions. Cen = centromere, RNA pol II = RNA polymerase II. **(B)** Representative pictures of TERRA RNA-FISH in cell nuclei (blue) from control (ctrl), tumor (RAS) and RNase treated tumor cells (+RNAse). TERRA foci are shown in magenta. Scale bar: 5 μm. **(C)** Scatter plot of TERRA RNA-FISH expression measured as the number of foci per nucleus in Control (Ctrl, *n* = 55) and tumor (RAS, *n* = 51) nuclei. Representative data from one of three experiments are shown (**p = 0.013). Bars represent mean ± SEM. **(D)** Scatter plot of TERRA RNA-FISH signals measured as fluorescent intensity per spot per nucleus in control (Ctrl) and RAS brains. Representative data from one of three experiments are shown (***p < 0.001). Bars represent mean ± SEM. **(E)** TERRA expression measured by dot blot from total RNA (500 ng) of a control and a RAS brain tumor (upper panel); to control for RNA loading, hybridization with an *actin* RNA probe was performed (lower panel). **(F)** qPCR analysis of TERRA expression in brain tumors and controls. Values were normalized first to *rps11* mRNA levels and then related to TERRA expression in control brains (*n* = 3). TERRA expression levels were also evaluated in HeLa and U2OS cells (*n* = 2 samples each) for comparison. Reactions without reverse transcriptase were performed as controls for TERRA quantification (-RT, ND: not detectable). Control bars represent mean ± SEM.

We could not asses the presence of ALT by probing for complexes of ALT-associated promyelocytic leukaemia (PML) bodies (APBs), due to the absence of a zebrafish ortholog of the PML gene (Carneiro et al., 2016). ALT cancer cells express higher levels of the telomeric lncRNA TERRA than telomerase-positive cancer cells (Lovejoy et al., 2012; Episkopou et al., 2014). We investigated the expression of TERRA, which is known to localize to ALT-associated PML bodies (Arora et al., 2014). TERRA is an RNA polymerase II transcript produced from subtelomeric regions toward chromosome ends (Azzalin and Lingner, 2015) (Figure 4A). To investigate TERRA expression, we performed RNA-FISH using TERRA-specific locked nucleic acid (LNA) probes in control and tumor cells (Figure 4B). By this analysis, we observed a significant increase in TERRA foci in tumor cell nuclei versus controls (Figures 4C,D). The signal was abrogated by RNase treatment, confirming the specificity of the assay (Figure 4B, +RNase). In order to confirm these results, we performed RNA dot-blot analyses on total RNA extracted from RAS or control samples. Here too, the signals were abrogated by RNase treatment, confirming the specificity of the assay (Supplementary Figure 4, +RNase). These experiments confirmed an increase in TERRA levels in tumors as compared to control brains (Figure 4E), which is consistent with the RNA-FISH results. Finally, TERRA expression levels were quantified by performing quantitative reverse transcription PCR using total RNA obtained from control and brain tumors and compared to TERRA levels in HeLa and U2OS cells. By these analyses, we found that TERRA levels in tumors were almost 3-fold higher than in control brains (Figure 4F, left panel). As expected, TERRA levels were found higher in U2OS cells as compared to the telomerase-positive HeLa cells (Figure 4F, right panel). Overall, these findings indicate that zebrafish brain tumors develop ALT and express high levels of TERRA.

**FIGURE 5:**
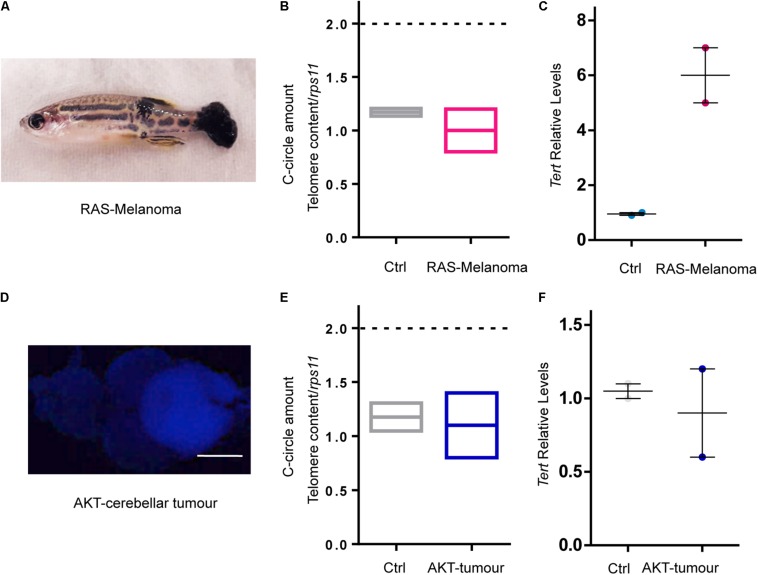
RAS expression is not the main driver of ALT in zebrafish cancer. **(A)** Image of a fish with melanoma induced by overexpression of oncogenic RAS. **(B)** C-Circle quantification by telomeric qPCR in control skin and melanoma (*n* = 2). **(C)** Measurement of tert expression in control skin and melanoma. Values were normalized first to *rps11* mRNA and then to *tert* expression in control skin (*n* = 2). **(D)** Image of a zebrafish brain with an AKT – driven cerebellar tumor. Calibration bar: 0.5 mm. **(E)** C-Circle quantification by telomeric Q-PCR in control brain and AKT – driven cerebellar tumors (*n* = 2). **(F)** Measurement of *tert* expression in control brains and AKT – driven cerebellar tumors. Values were normalized first to *rps11* and then to *tert* expression in control brains (*n* = 2). Bars represent mean ± SEM.

As in this zebrafish model, brain tumors are induced by the expression of the human oncogene RAS, we asked whether RAS expression may induce ALT in zebrafish. To address this question, we used a different zebrafish model of cancer in which tissue-specific expression of RAS induces melanoma (Santoriello et al., 2010; Figure 5A). Detailed analyses of ALT hallmarks revealed that ALT did not develop in this model, in which the levels of tert expression were detected as fivefold higher than in control skin (Figures 5B,C). These results indicate that RAS expression is not a general driver of ALT in zebrafish cancer.

Similarly, we tested if ALT could be the preferred TMM of zebrafish brain cells when they form tumors. We generated cerebellar tumors using myristylated AKT (AKT) under the control of the zic:Gal4 promoter as described in Mayrhofer et al. (2017) (Figure 5D) and we observed that neither ALT activity nor *tert* gene expression was changed compared to control cerebellar tissue (Figures 5E,F). Cerebellar tumors induced by AKT overexpression arise sporadically, mainly after 4 months (data not shown), when the fish are adults, as opposed to the RAS induced tumors that start to develop at 3 dpf. Thus, the brain tumor model generated through the overexpression of oncogenic RAS in neural progenitors is the first *in vivo* GEM model of ALT brain tumors, and this feature is not due to the RAS oncogene but may be related to the early development of brain tumors in the RAS model.

### A Reduction of *tert* Expression Precedes the Development of ALT

**FIGURE 6:**
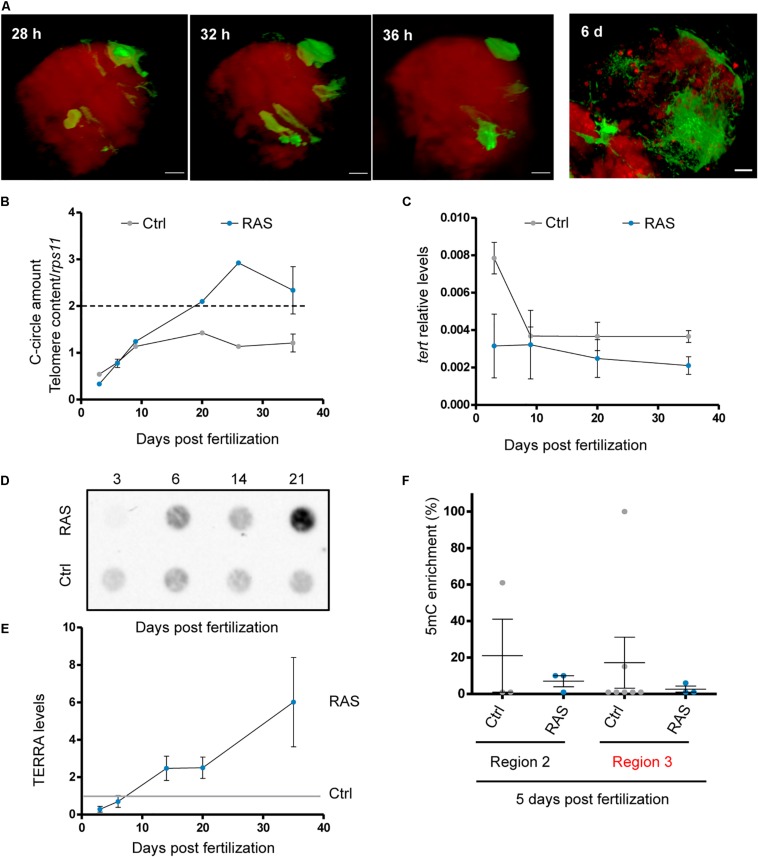
Development of ALT is preceded by a reduction of *tert* expression. **(A)** Representative images of tumor development from single cancer initiating clones to tumoral masses. h: hours; d: days. **(B)** C-Circle assay measured by telomeric qPCR during tumor development in control and RAS brains. Data are represented as CCA amount normalized to telomere content (TC) and single copy gene (*rps11*). The dashed line indicates the level above which ALT activity is considered significant Bars represent mean ± SEM. **(C)** RT-qPCR analysis of tert expression during tumor development. The data were normalized first to rps11 mRNA levels and are expressed as 2 (–ΔCt). Bars represent mean ± SEM. The experiment was replicated almost three times for each time points. **(D)** Representative dot blot of TERRA levels during tumor development (500 ng of total RNA was spotted for all samples) and **(E)** quantification of three independent experiments. Background was removed and values were normalized to the levels of TERRA in controls of the same larval stages (gray line). F) qPCR analysis of DNA methylation (5-methylcytosine, 5mC-ChIP) status of the tert promoter in 5 dpf control (*n* = 3–5) and RAS (*n* = 3) fish larvae. Two regions of the promoter (see Figure 2C) were analyzed, the red color of region 3 indicates a putative CpG island. Values were normalized first to *rps11* and then to 5mC vs. IgG enrichment, which is set at 1.0.

The model of brain tumor presented here allows the study of progression from a single cancer-initiating cell to a full tumor (Figure 6A). We investigated when ALT was activated during the progression of brain cancer, by performing CCA from the early stages (3 dpf) to full tumor development (1 m). This time interval corresponded to the entire larval period during which tumors grow progressively. In these experiments, we observed the presence of C-Circles above control levels starting from 20 dpf (Figure 6B). During the same period, we also studied the changes in *tert* expression (Figure 6C) and TERRA levels (Figures 6D,E). Lower levels of *tert* expression compared to control brains of the same age were observed throughout tumor development, and a further decrease of *tert* levels preceded the increase of C-Circles at 20 dpf (Figure 6C compared to 6B). In particular, *tert* expression levels decrease in brains developing tumors to reach 1.7-fold less than controls at 1 m (Figure 6C), when the tumors were fully formed.

Furthermore, TERRA levels positively correlate with ALT activity, and an increase of TERRA expression was already detected at 14 dpf (Figures 6D,E), just before the increase in C-Circle levels.

We then examined the methylation status of the *tert* promoter at 5 dpf. We considered two of the most representative regions upstream of the transcriptional start site of the *tert* gene (Figure 2C, regions 2 and 3). We found hypomethylation of the *tert* promoter in RAS compared to controls already at 5 dpf (Figure 6D), suggesting that the expression of *tert* in the brain was negatively modulated from the first days of RAS expression. Moreover, we found abnormalities in telomere signals in metaphases already at 5 dpf (Supplementary Figures 2A,B, yellow arrows).

In summary, these results suggest that a reduction of *tert* expression and an up-regulation of TERRA levels precede the activation of ALT.

### Expression of Functional *tert* Prolong Survival of Fish With Brain Tumors

**FIGURE 7:**
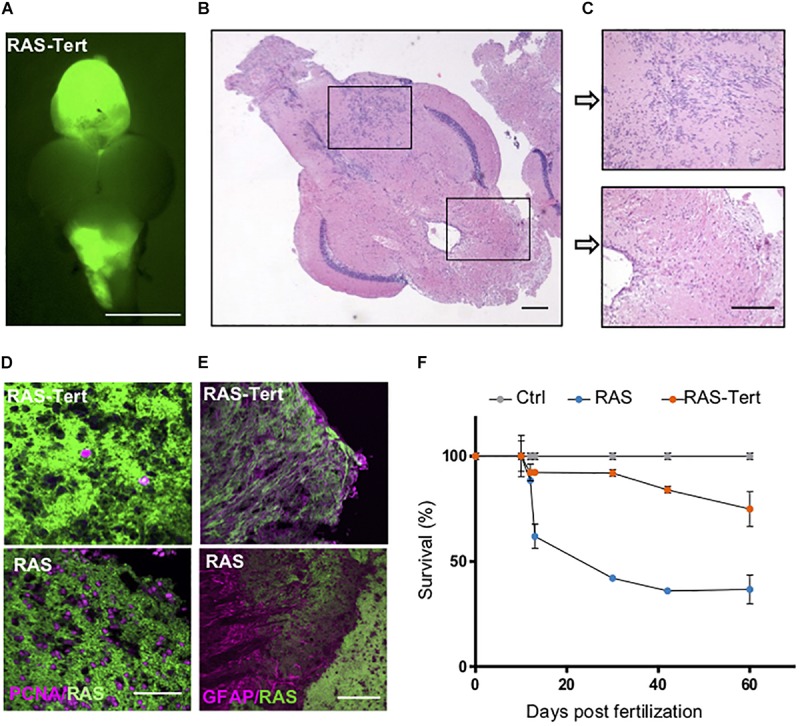
Expression of functional *tert* prolongs survival of fish with brain tumors. **(A)** Representative fluorescent image of a RAS-Tert brain tumor. Scale bar 0.5 mm. **(B)** Histological analysis of the RAS-Tert brain tumor shown in panel **(A)**, **(C)** magnification of two area showing mild neoplastic abnormalities. Scale bars: 20 μm. **(D)** Immunofluorescence images showing the distribution of the proliferation marker PCNA (magenta) and **(E)** of the differentiation marker GFAP (magenta) in sections of tumors from RAS-Tert and RAS brains. Scale bar: 20 μm. **(F)** Survival curve during the entire larval period up to 2 m of Control, RAS and RAS-Tert fish (*n* = 45–60 larvae/genotype in three independent experiments). See also Supplementary Figure 3.

To evaluate if ALT activation could be prevented by maintaining high levels of telomerase, we increased *tert* and *terc* levels with transgenesis. To this aim, we first generated two stable transgenic lines, *tg(10xUAS:tert)* and *tg(10xUAS:terc)* (Supplementary Figures 3A,C). Then we generated a triple transgenic line were *tert*, and *terc* were overexpressed in neural progenitor cells (zic:Gal4 + cells) and we induced the development of brain tumors by injection of oncogenic RAS at one-cell stage. The brain tumors, indicated as RAS-Tert (Figure 7A), were compared with RAS (only) tumors using several parameters (see below).

The overexpression of *tert* in the RAS-Tert line was verified through RT-qPCR using primers designed between the 12 and 13 exons of the tert transcript (Supplementary Figure 3D) and within the 3′UTR for the endogenous *tert* levels (Supplementary Figure 3E), to distinguish total *tert* from endogenous *tert* levels. Total *terc* levels were not found increased compared to control fish as detected by RT-qPCR (Supplementary Figure 3F).

We observed that the formation of neoplastic lesions due to induction of oncogenic RAS (Figure 7B) occurred with the same frequency in RAS-Tert and in RAS fish, being 100% in both cases, and in similar locations. In particular, in both genetic backgrounds, brain tumors appeared at 3–4 weeks in the telencephalon, IV ventricle and diencephalon (Figure 7A). Immunohistological analysis of several RAS-Tert brain tumors showed that they were less proliferative (Figure 7C, upper panel) and more differentiated, expressing the glial fibrillary acidic protein, GFAP (Figure 7D, upper panel) than those that developed in RAS fish.

Even though both RAS and RAS-Tert fish developed tumors with a similar frequency and timing, the two models differed significantly in overall survival, with the RAS-Tert fish showing 83.97% survival at 2 months whereas only 36.9% RAS fish survived by the same time (Figure 7E). These findings suggest that tumors developed in RAS-Tert fish are less aggressive than tumors arising in RAS fish.

### Expression of *tert* Prevents the Development of ALT and Promotes Telomere Integrity Through Heterochomatin Maintenance in Brain Tumors

**FIGURE 8:**
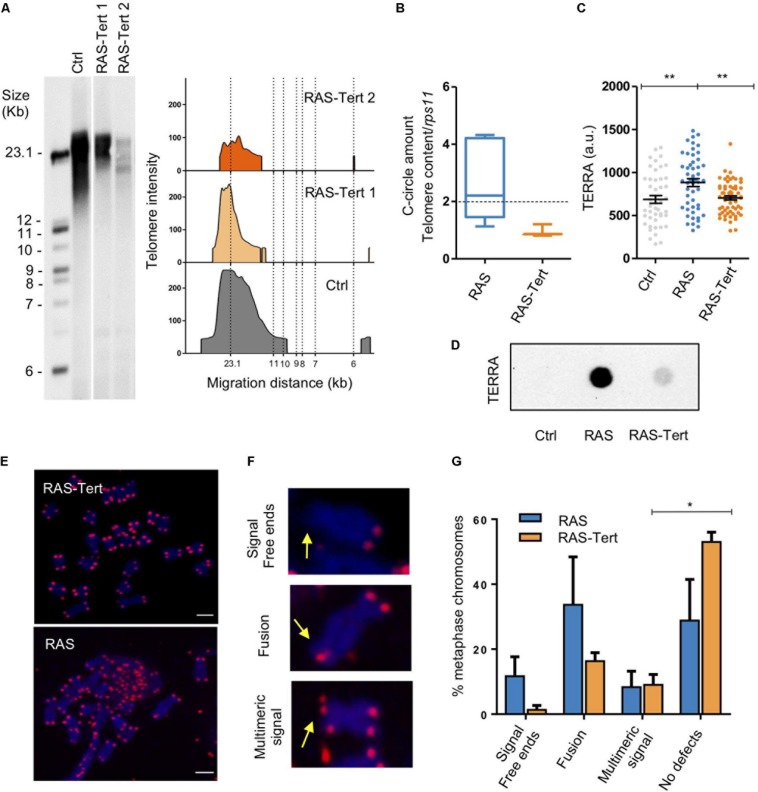
Expression of *tert* prevents the development of ALT and reduce genome instability in brain tumors. **(A)** Telomere length analysis by TRF in one control and two RAS-Tert tumors. The panel on the right shows TFR analysis obtained by graphing intensity versus DNA migration. The southern blot was sliced for better fitting. **(B)** C-Circle assay quantified by telomere qPCR in RAS and RAS-Tert tumors. Data are represented as C-Circle amount normalized to telomere content (TC) and single copy gene (*rsp11*). RAS *n* = 7; RAS-Tert *n* = 3. The dashed line indicates the level above which ALT activity is considered significant. Whiskers box plots represent median: min to max values. **(C)** Scatter plot of TERRA RNA-FISH signals measured as fluorescent intensity of single spots per nucleus in Control (*n* = 46), RAS (*n* = 50), and RAS-Tert (*n* = 62) cell nuclei from brains and brain tumors. Data from a single, representative experiment are shown (**p < 0.01). Bars represent mean ± SEM. **(D)** TERRA RNA dot blot from total RNA of control, RAS and RAS:Tert brains. 500 ng of total RNA was spotted for all samples. **(E)** Metaphase DNA FISH analysis using telomeric repeat probes of Tert-RAS and RAS juvenile brain tumors. Telomeric DNA (magenta), metaphase chromosomes, blue, and **(F)** magnified examples of aberrant telomere phenotype, signed by arrows: Signal free ends, fused telomeres, multimeric signal. **(G)** Quantitation of telomeric defects revealed in RAS (*n* = 95 chromosomes) and RAS-tert (*n* = 68 chromosomes) tumors. Multiple *t*-test, Holm Sidak methods, *p < 0.05. Bars represent mean ± SEM.

Next, we evaluated telomere length and the presence of C-Circles in RAS-Tert tumors and found that telomere length was very similar to that of control brain cells with a substantial reduction in telomere length heterogeneity (Figure 8A). The assay repeated with a longer telomeric probe shows a TRF signal in RAS-Tert brain tumors typical of telomerase + cells (Supplementary Figure 3G). CCA products were also reduced to the levels detected in control brains (Figure 8B). Moreover, the levels of TERRA in RAS-Tert tumors were comparable to TERRA levels detected in non-tumoral samples (Figures 8C,D). These findings suggest that the re-expression of *tert* prevented ALT development and TERRA increase in zebrafish brain tumors.

We also determine that expression of *tert* reduced certain telomeric defects present in ALT brain tumors (Figures 8E–G). DNA FISH analysis of metaphase spreads revealed a lower occurrence of signal free ends and fusion events on sister chromatids; by contrast, no difference in the occurrence of multimeric signals between the two genotypes was found (Figures 8E–G).

Together, these data point to a role of tert in preventing ALT development and TERRA increase, and most importantly in promoting telomeric stability during tumor development.

To investigate how the expression of *tert* in zebrafish brain tumors promotes genome stability, we evaluated the levels of heterochromatin at telomeres. We performed immunostaining for H3K9me3 in combination with Q-FISH, in RAS and RAS-tert tumors. In addition, we evaluated telomeric DNA damage using γH2AX immunostaining in conjunction with Q-FISH.

**FIGURE 9:**
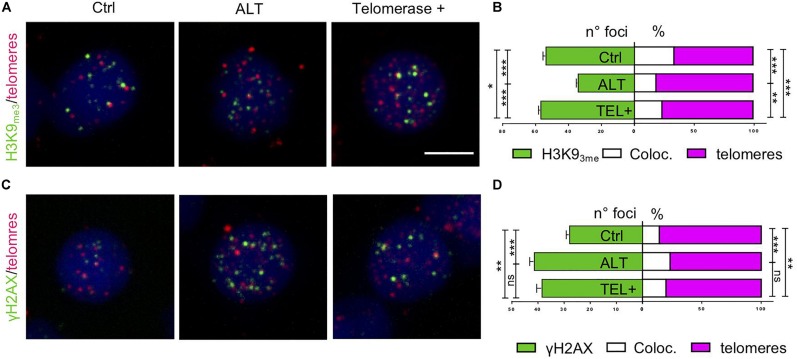
Expression of *tert* maintains telomeric heterochromatin in brain tumors. **(A,C)** Fluorescent microscope images of representative control, ALT, and telomerase + zebrafish brain tumor cells, stained via immunofluorescence (green) combined with telomere-FISH (magenta). Antibody against chromatin methylation marks (**A**, H3K9me3) and DNA damage marker (**C**, γH2AX), were used and counterstained with DAPI. Scale bar: 5 μm. **(B,D)** Immunofluorescence quantification expressed as the number of foci per nucleus (green), and percent of immunofluorescence foci (white) that colocalized at telomeres (magenta) per nucleus (*n* = 25–60 nuclei) for the corresponding images in panel **(A,C)**. *p < 0.05, ***p < 0.001 between the indicated groups. Bars represent mean ± SEM.

These experiments showed that there is an increase of H3K9 methylation at the level of telomeres in RAS-tert tumor cells compared to RAS tumors (Figures 9A,B). In addition, telomeric DNA damage was reduced in RAS-tert compared to RAS tumors, although not significantly (Figures 9C,D).

These observations suggest that re-expression of *tert* in brain tumors restores heterochromatin and reduces the occurrence of telomeric DNA damage. These events may lead to the better survival of fish with *tert* positive brain tumors.

## Discussion

During recent years, the zebrafish has been used to study telomere biology in relation to organismal aging using mostly the telomerase mutant *hu3430* (Anchelin et al., 2011, 2013; Henriques et al., 2013; Carneiro et al., 2016). Here, we characterize the TMM used by tumor cells in a zebrafish model of brain cancer, which highly resembles human pediatric glioblastoma of mesenchymal origin, a very aggressive tumor associated with poor prognosis. We found that zebrafish tumors rely on ALT as TMM, similarly to many pediatric GBMs. With the increasing number of reports documenting a prevalence of ALT in some pediatric cancers (Heaphy et al., 2011; Dorris et al., 2014; Deeg et al., 2017) the role of the embryonic origin of these tumors in the ability to activate telomerase or ALT during cancer progression is becoming evident.

By investigating the RAS zebrafish cancer model we report that ALT can also develop as a consequence of the lack of telomerase expression during tumor development. Indeed, sustained expression of *tert* in the neural progenitors that initiate tumorigenesis prevented ALT development. Previous evidence in human cancer cell lines have shown that expression of hTERT does not abolish ALT (Perrem et al., 2001). However, genetic ablation of telomerase in a mouse cancer model can lead to ALT development (Cesare and Reddel, 2010), suggesting that telomerase activity can prevent the emergence of ALT in cancer.

Recent studies investigated is the epigenetic regulation of telomerase activation in cancer. Lee et al. (2019) identified a specific region within the TERT promoter, termed upstream of the transcription start site (UTSS), that is frequently hypermethylated in TERT-expressing cancers, suggesting an association between TERT promoter hypermethylation and elevated TERT expression in cancer. In our model, we found a general hypomethylation of the tert promoter in brain tumor cells that are prone to activate ALT, suggesting that oncogenic events taking place during early developmental stages influence the methylation status of the tumor-initiating/propagating cells that generate the tumors. Accordingly, epigenetic alterations can influence cell identity and TMMs activation. Indeed, tumor cells with stem cell-like properties have been identified in a wide range of pediatric brain cancers (Hemmati et al., 2003; Galli et al., 2004).

Several reports suggest the presence of telomeres with reduced levels of heterochromatin in ALT cells compared to telomerase-positive cells (Episkopou et al., 2014; Pickett and Reddel, 2015). The open chromatin status may lead to the incorporation of non-canonical variant repeats, which alter the binding of the shelterin complex and reinforce telomere de-protection. Disruption of the telomeric chromatin environment results in higher levels of TERRA transcription. TERRA transcripts may participate in ALT induction by multiple mechanisms: through formation of DNA:RNA hybrids, or R-loops, which may promote homologous recombination among telomeres (Arora et al., 2014); by interfering with ATRX functions, as described in mouse embryonic stem cells (Chu et al., 2017); or by impacting replication of telomeric DNA (Beishline et al., 2017). Moreover, TERRA has been shown to regulate directly telomerase activity in embryonic stem cells. Chu et al. (2017) observed a twofold upregulation of telomerase activity following TERRA knock down. In the zebrafish brain cancer model, sustained telomerase expression in tumor initiating cells not only prevents ALT, but also represses TERRA transcription. These observations establish that TERRA and telomerase play antagonistic roles in TMMs by multiple mechanisms, since the re-expression of *tert* in brain tumors restores heterochromatin and reduces the expression of TERRA. These events improve fish survival and pave the way for future therapeutic intervention in ALT brain tumors.

Why is ALT more frequent (and prognostically worst) in pediatric tumors? It is plausible that reactivation of telomerase, which is usually achieved in cancer through mutations of the TERT promoter that hinder a repressor binding site, may require time for selection of the right mutation; therefore a quicker, although dangerous, mechanism to maintain telomere length and allow cancer growth, is through the engagement of ALT mechanisms. In addition, young cells, such those in developing brains or bones (for sarcomas), have probably not yet completely organized their heterochromatin, so that their hypomethylated chromatin environment makes it easier to switch to ALT. The mechanism is similar in the frequent mutations in H3.3 and ATRX in pediatric brain cancer, which compromise chromatin structure and promotes genomic instability and ALT.

## Data Availability Statement

All datasets generated for this study are included in the article/Supplementary Material.

## Ethics Statement

The animal study was reviewed and approved by the D.Lgs. 26/2014, authorization 148/2018-PR to MM.

## Author Contributions

AI and MM conceived the study and performed all the experiments and the analyses, with the exception of the *tert* promoter ChIP experiments, that were performed by EC. FP and PP performed the histological analysis of the zebrafish brain tumors. MB and MC performed the generation of the *tg(10xUAS:tert)* and *tg(10xUAS:terc)* zebrafish transgenic lines. FB guided the optimization of the Q-FISH, 2-color CO-FISH and metaphase preparations. AI, EC, and MM wrote the manuscript. All authors commented on the manuscript.

## Conflict of Interest

The authors declare that the research was conducted in the absence of any commercial or financial relationships that could be construed as a potential conflict of interest.

## Footnotes

1 http://tol2kit.genetics.utah.edu/

2 https://imagejdocu.tudor.lu/doku.php?id=plugin:analysis:distance_analysis_diana_2d_3d_:start

3 https://www.ebi.ac.uk/Tools/seqstats/emboss_cpgplot/

4 https://www.ensembl.org/Danio_rerio/
