# Supplementary material for: Expression of tert Prevents ALT in Zebrafish Brain Tumors
Source: Front Cell Dev Biol. 2020 Feb 11;8:65. doi: 10.3389/fcell.2020.00065 (PMC7026139; doi:10.3389/fcell.2020.00065)
Supplement: Supplementary file 1 [file Presentation_1.PPTX]

## Slide 1
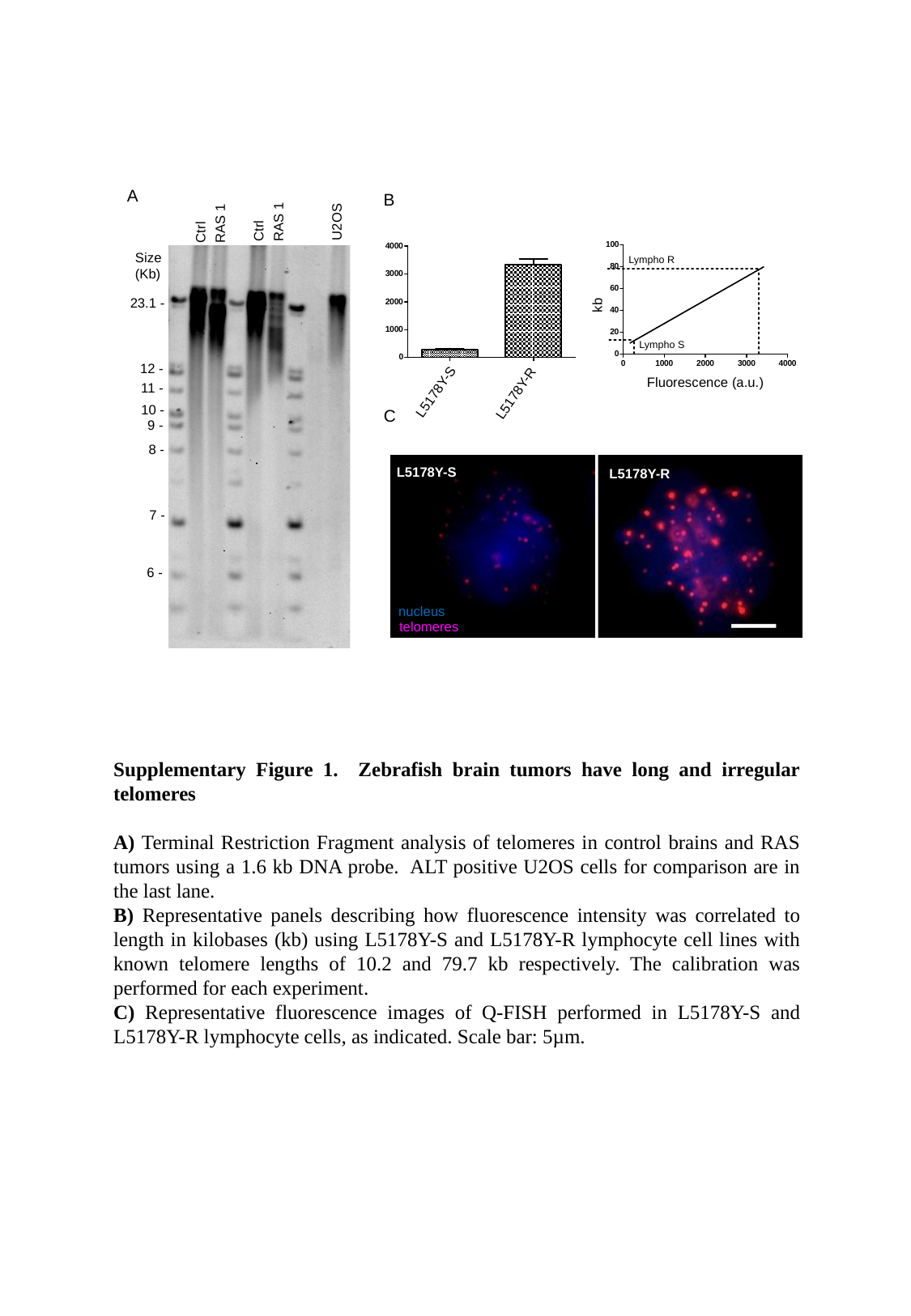

A
B
U2OS
RAS 1
RAS 1
Ctrl
Ctrl
Lympho R
kb
Lympho S
Fluorescence (a.u.)
L5178Y-S
L5178Y-R
Size
(Kb)
23.1 -
12 -
10 -
9 -
7 -
6 -
11 -
8 -
C
L5178Y-S
nucleus
telomeres
L5178Y-R
Supplementary Figure 1. Zebrafish brain tumors have long and irregular telomeres
A) Terminal Restriction Fragment analysis of telomeres in control brains and RAS tumors using a 1.6 kb DNA probe. ALT positive U2OS cells for comparison are in the last lane.
B) Representative panels describing how fluorescence intensity was correlated to length in kilobases (kb) using L5178Y-S and L5178Y-R lymphocyte cell lines with known telomere lengths of 10.2 and 79.7 kb respectively. The calibration was performed for each experiment.
C) Representative fluorescence images of Q-FISH performed in L5178Y-S and L5178Y-R lymphocyte cells, as indicated. Scale bar: 5µm.
Zic Ras
Zic Ras

## Slide 2
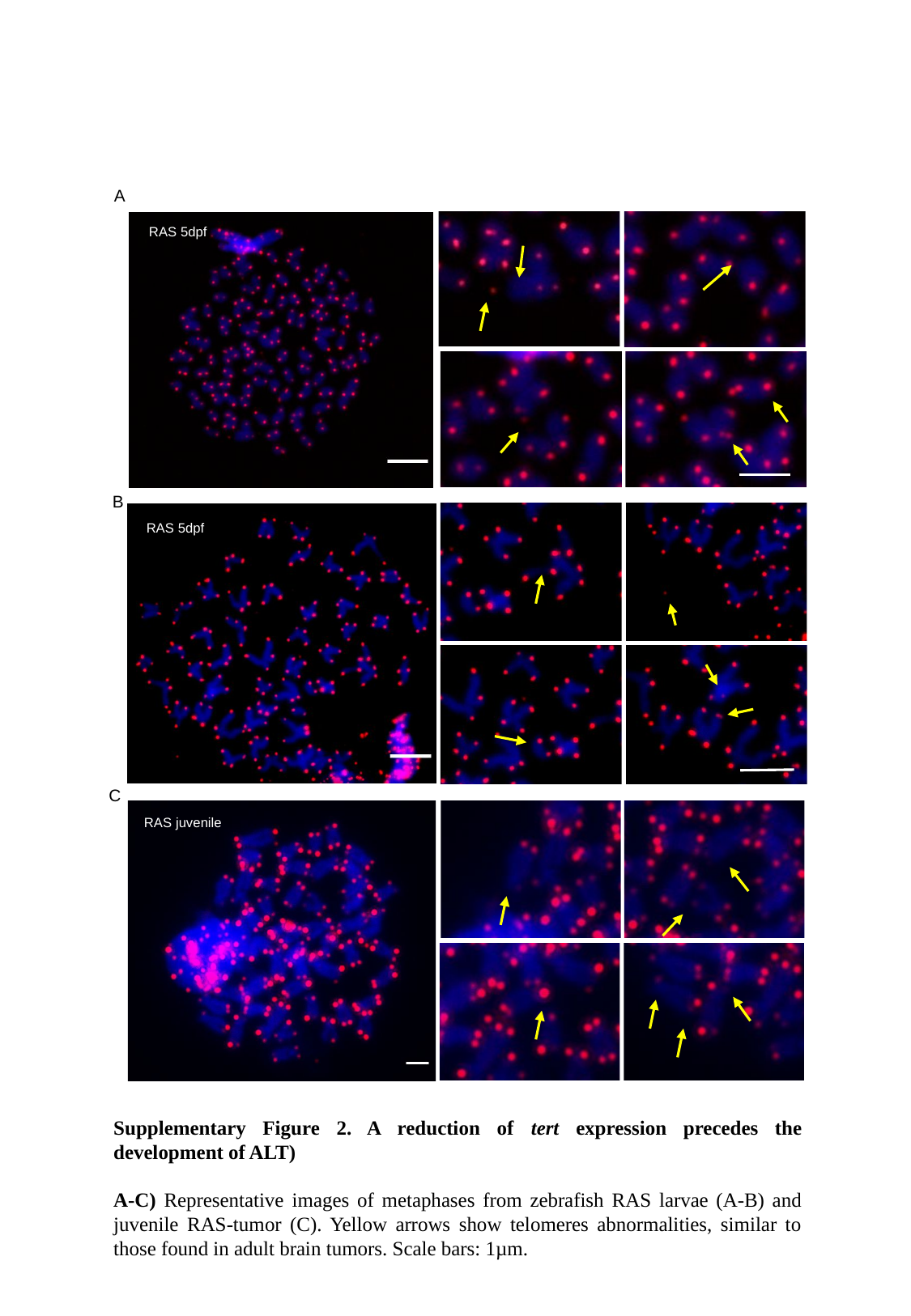

A
RAS 5dpf
B
RAS 5dpf
C
RAS juvenile
Supplementary Figure 2. A reduction of tert expression precedes the development of ALT)
A-C) Representative images of metaphases from zebrafish RAS larvae (A-B) and juvenile RAS-tumor (C). Yellow arrows show telomeres abnormalities, similar to those found in adult brain tumors. Scale bars: 1µm.

## Slide 3
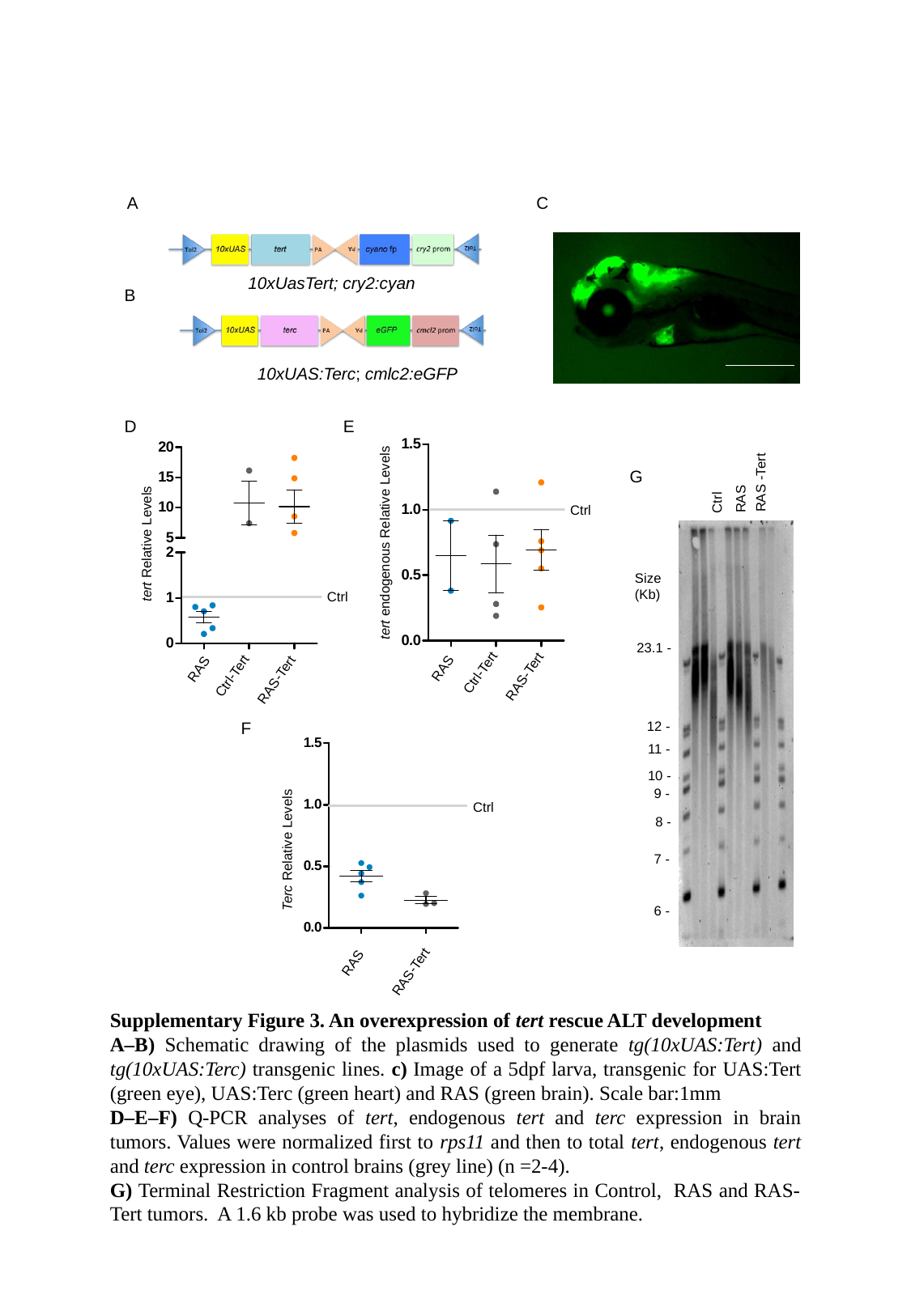

C
A
10xUasTert; cry2:cyan
B
10xUAS:Terc; cmlc2:eGFP
D
E
tert endogenous Relative Levels
RAS
Ctrl-Tert
RAS-Tert
tert Relative Levels
Ctrl
RAS
Ctrl-Tert
RAS-Tert
RAS -Tert
RAS
Ctrl
Size
(Kb)
23.1 -
12 -
10 -
9 -
7 -
6 -
11 -
8 -
G
Ctrl
F
Ctrl
Terc Relative Levels
RAS
RAS-Tert
Supplementary Figure 3. An overexpression of tert rescue ALT development
A–B) Schematic drawing of the plasmids used to generate tg(10xUAS:Tert) and tg(10xUAS:Terc) transgenic lines. c) Image of a 5dpf larva, transgenic for UAS:Tert (green eye), UAS:Terc (green heart) and RAS (green brain). Scale bar:1mm
D–E–F) Q-PCR analyses of tert, endogenous tert and terc expression in brain tumors. Values were normalized first to rps11 and then to total tert, endogenous tert and terc expression in control brains (grey line) (n =2-4).
G) Terminal Restriction Fragment analysis of telomeres in Control, RAS and RAS-Tert tumors. A 1.6 kb probe was used to hybridize the membrane.

## Slide 4
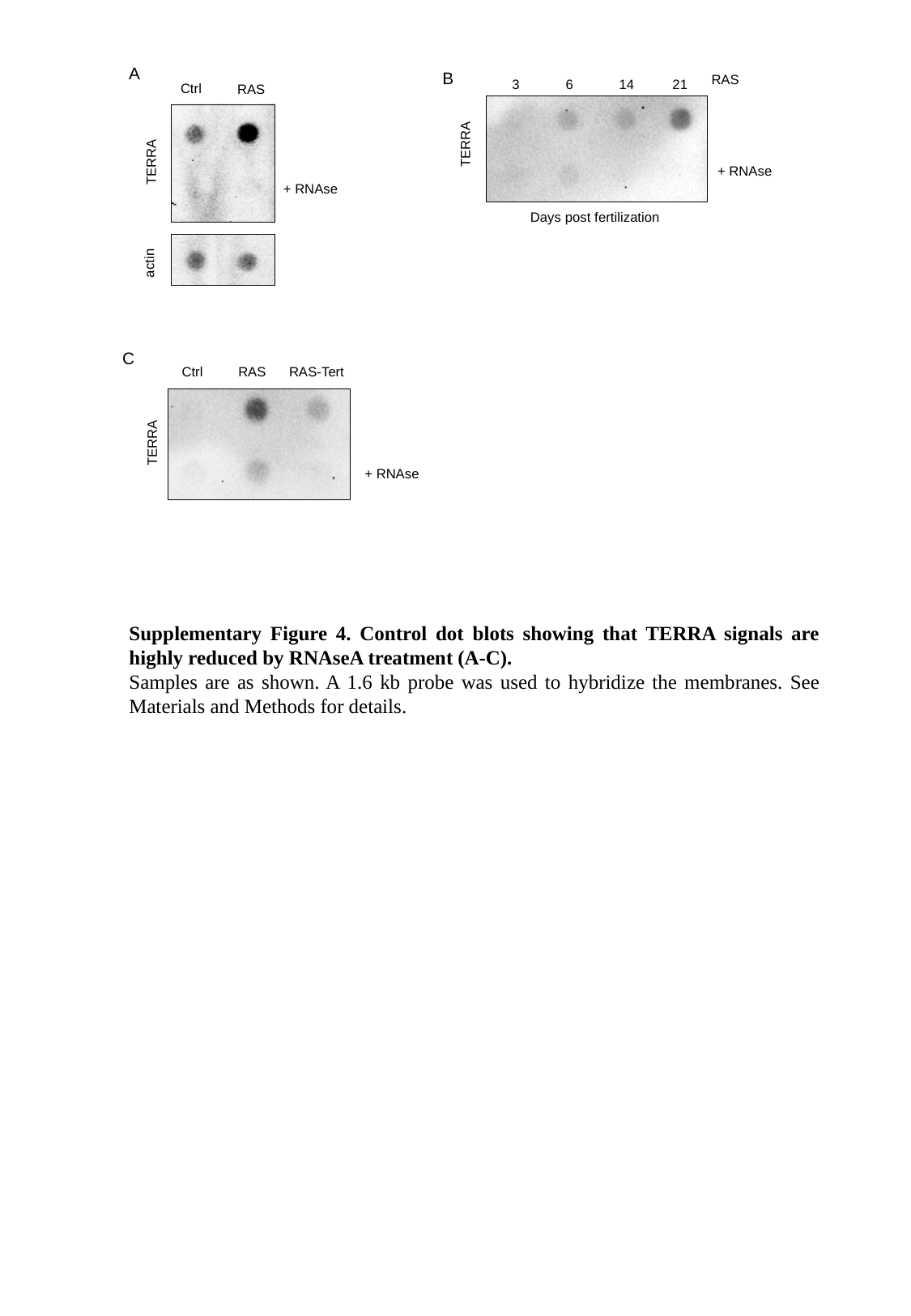

A
B
RAS
3 6 14 21
Ctrl
RAS
TERRA
TERRA
+ RNAse
+ RNAse
Days post fertilization
actin
C
Ctrl
RAS
RAS-Tert
TERRA
+ RNAse
Supplementary Figure 4. Control dot blots showing that TERRA signals are highly reduced by RNAseA treatment (A-C).
Samples are as shown. A 1.6 kb probe was used to hybridize the membranes. See Materials and Methods for details.
